# Saturating effects of species diversity on life-history evolution in bacteria

**DOI:** 10.1098/rspb.2015.1794

**Published:** 2015-09-22

**Authors:** Francesca Fiegna, Thomas Scheuerl, Alejandra Moreno-Letelier, Thomas Bell, Timothy G. Barraclough

**Affiliations:** Department of Life Sciences, Imperial College London, Silwood Park Campus, Ascot, Berkshire SL5 7PY, UK

**Keywords:** species diversity, evolution, microbial, life history

## Abstract

Species interactions can play a major role in shaping evolution in new environments. In theory, species interactions can either stimulate evolution by promoting coevolution or inhibit evolution by constraining ecological opportunity. The relative strength of these effects should vary as species richness increases, and yet there has been little evidence for evolution of component species in communities. We evolved bacterial microcosms containing between 1 and 12 species in three different environments. Growth rates and yields of isolates that evolved in communities were lower than those that evolved in monocultures, consistent with recent theory that competition constrains species to specialize on narrower sets of resources. This effect saturated or reversed at higher levels of richness, consistent with theory that directional effects of species interactions should weaken in more diverse communities. Species varied considerably, however, in their responses to both environment and richness levels. Mechanistic models and experiments are now needed to understand and predict joint evolutionary dynamics of species in diverse communities.

## 1. Introduction

Most studies of contemporary evolution consider focal species. This approach provides great insights into genetic mechanisms, the effects of fluctuating environments and trait correlations. However, all species live in diverse communities of many hundreds or more species. If ecological interactions alter selection on constituent species in communities, then the magnitude and direction of evolution might change as diversity increases [1–5]. The amount of evolution might decrease in species-rich communities, because ecological interactions limit ecological opportunity for traits to evolve [6,7]. However, interactions might also promote evolution through coevolution or by strengthening selection caused by abiotic conditions [2,8]. Despite being important for understanding evolution in the wild [9], general effects of diversity on evolution of all species in a community are hard to investigate. There have been few studies comparing different species evolving in the same environment [10].

Here, we investigate the effects of species richness on life-history evolution in bacterial communities cultured in the laboratory. A wealth of theory and evidence predicts changes in monocultures in serial transfer conditions typical of evolution experiments, as follows. If selection pressures are too strong, the species will fail to adapt and dwindle to extinction [11]. Otherwise, growth rate (*r*) should increase, because selection favours fast growth from low densities after each serial transfer event [12]. The increase in growth rate should be greater in species with initially lowest growth rates [12] and in new environments, causing a greater initial decline in growth rate (as long as growth rates are not below the threshold for extinction). The maximum density or yield in turn might be expected to decline if growth rate increases if there are negative mechanistic trade-offs between growth rates and yield [13–15]. These predictions assume that serial transfers occur often enough to maintain the population in exponential growth phase. If instead the interval between transfer events is long enough for density to limit growth rates (i.e. population density approaches its maximum), then species should evolve to attain higher yield at the time of transfer (for example through greater efficiency of resource use). Although phrased in terms of microbial evolution in serial transfer, the above predictions apply widely to populations growing with ongoing input and depletion of resources, such as wild animal populations [16] or even cancer cells [17].

Species interactions in communities might alter predictions in several ways. If species interactions are mostly negative [18], then extinction rates should increase as growth rates of some species are depressed below the threshold enabling survival. Measuring growth of surviving species extracted from the communities they evolved in, growth rates might be reduced compared with isolates that evolved in monocultures if competition limits the opportunity for species to specialize on those resources supporting fastest growth [6,19,20]. However, growth rates of some species might increase relative to monocultures if competition drives the evolution of enhanced competitive ability on shared resources [8]. Alternatively, if there is a negative trade-off between growth rate and yield, different species might diverge and specialize to either high-rate or high-yield strategies [15,21,22]. Finally, there might be no effect on evolution. The balance of these different effects might shift as species richness increases.

Previous studies have shown that species interactions do affect life-history evolution. Guppy life history evolves along an environmental gradient from a single predator to diverse predatory fish communities [23]. Similarly, selection on life history of a marine bryozoan was altered by the intensity of interspecific competition by presence or absence of other colonizing organisms [24]. terHorst [25] found that growth rates and peak densities of protists evolved in the presence of either a predator (mosquito larvae) or competitor (another protist), but that the presence of both inhibited evolution. However, multigenerational studies are needed that manipulate species diversity while controlling for other environmental features that might covary with diversity in the wild. As diversity increases, strong pairwise interactions might become less important relative to numerous diffuse interactions or act in opposing directions, so that the effects of species interactions on evolution saturate or reverse in more diverse communities [26].

**Figure 1.**
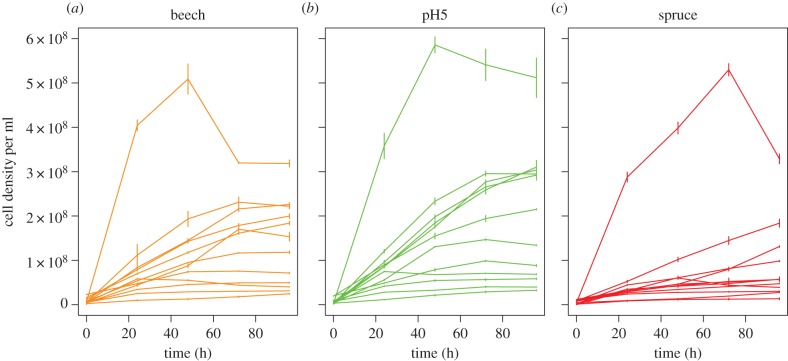
Growth curves of calibrated cell density per ml of ancestral isolates of each species over 96 h on the three experimental media: (*a*) beech-leaf tea, (*b*) pH5 beech tea and (*c*) spruce tea. Curves show the averages across three replicates per species and standard errors are shown. Malthusian growth rates over the first 24 h did not vary significantly among environments (*F*_2,105_ = 1.25, *p* = 0.29). Maximum yields did vary significantly among environments (*F*_2,105_ = 4.13, *p* = 0.019), being marginally higher in pH5 and lower in spruce tea.

We tested these ideas using artificial communities of up to 12 species of bacteria isolated from small pools formed by the roots of beech trees. All species were obligate or facultative aerobic heterotrophs. Microcosms with 1, 2, 3, 6 or 12 species were then cultured with serial transfer for around 60 generations on three environments: a control environment on beech-leaf tea medium; a ‘benign’ environment of more acidic pH5 beech tea that ancestral isolates grew to higher yield on than control tea; and a ‘harsh’ environment of spruce tea that ancestral isolates grew to a lower yield on than control tea (figure 1). Note that these environments probably varied in multiple factors, such as pH and chemical composition of resources, including carbon and nitrogen availability. We do not focus on the effects of specific factors here, however, but simply chose two alternative environments that had different effects on ancestral growth rates that were potentially relevant to real tree-holes.

Previously, we investigated how species interactions and ecological functioning evolved over time by comparing monoculture and community yields [27]. Here, we compare initial growth rate from low density (*r*) and yield (maximum density) between ancestral isolates and final isolates from each environment and species richness treatment. We predicted that growth rates should increase and yields decline across monocultures as outlined above. The effects should be greatest in the ‘harsh’ spruce tea than in the ‘benign’ pH5 tea. The effects of species interactions on evolution of constituent species were evaluated by comparing growth rates and yields of surviving species from diverse communities with monoculture isolates of those species. Note that both community and monoculture isolates were assayed finally in their ability to grow in their experimental environment in the absence of other species: although this does not match the community context that they evolved in, it provides a tractable assay of changes in growth phenotypes that can be interpreted in the light of changes occurring in communities. We predicted that species isolated from communities should display lower growth rates and/or yields when cultured alone, if species interactions limit their ability to evolve the use of resources associated with high rate and/or yield, but that the strength of effect should saturate with increasing number of species.

## 2. Methods

### (a) Isolation of study species

We isolated bacteria from small pools formed by the roots of beech trees. Isolates were obtained by plating out aqueous samples from a tree-hole at Silwood Park, Ascot, UK on R2A agar and identified by 16S rDNA sequencing as described by Fiegna *et al*. [27]. All species were obligate or facultative aerobic heterotrophs. Twelve isolates were chosen that had different growth morphology and colour on agar plates to facilitate their isolation from mixed co-cultures at the end of the experiment (electronic supplementary material, table S1).

### (b) Evolution experiment

Microcosms were set up in the laboratory with, in turn, each species in monoculture, each species in two different compositions of two-, three- and six-species communities in turn in a random partitioned design [28,29], and all 12 species co-cultured together. There were therefore 12 monocultures plus 25 communities, giving 37 compositions in total (electronic supplementary material, table S2). Each composition treatment was replicated three times. Microcosms were cultured for 2 weeks in 10 ml of standard beech tea medium made by autoclaving 50 g of autumn fall beech leaves in 500 ml of water and diluted 32-fold. Cultures were set up using a substitutive design to equivalent optical densities. We added R2A ingredients [30] to increase growth rates and the number of generations during the experiment (details in [27]; the media included 0.375 g glucose and 0.374 g soluble starch per litre as the main added carbon, enough to speed up growth rates but such that most carbon still came from the beech leaves). Alternating every 3 or 4 days, 150 µl of culture was transferred to fresh medium to maintain active growth and maximize the number of generations. After this interval, some ancestral isolates were still increasing in density, whereas others had peaked already (figure 1) and therefore, in monocultures, we would expect selection for faster growth rates and/or higher yield depending on the species. After two weeks, we split each microcosm into three treatments: standard beech tea, beech tea with its pH lowered from 7 to pH5, and spruce tea made similarly but from spruce needles (total 333 microcosms). Phosphate buffers were used to maintain beech tea and spruce tea at pH7 and to obtain pH5 tea. Microcosms were cultured for six further weeks. To track community densities, we measured optical density (OD) at 595 nm every 24 h during the experiment. The number of doublings in monoculture ranged from 62 to 91 in beech tea, 60 to 87 in pH5 tea and 21 to 70 generations in spruce tea for each species (estimated as log base 2 of the ratio of final over starting density for each growth period, which assumes births but not deaths during each growth period). Final cultures were streaked out on agar plates and isolates picked off for surviving species. Where species expected to be present were absent, multiple plates were inoculated at multiple dilutions: we estimate that any species surviving at a density of 1 in 10 000 or more would typically have been recovered. Final identifications of species from colony morphology and colour were checked with 16S sequencing [25]. All isolates were stored in −80°C prior to growth assays.

### (c) Growth assays and statistical analyses

Frozen isolates of species recovered from each microcosm were grown up in 150 µl of their ‘home’ medium for growth assays (i.e. isolates that evolved in beech tea were assayed in beech tea; ancestral isolates were assayed in all three media). OD at 595 nm was measured every 24 h for 96 h to calculate growth rate from low density (*r,* estimated as log.OD at 24 h minus log.OD at 0 h divided by 24) and yield (estimated as the maximum density observed over the 96 h). We measured the growth of 972 isolates in total. A flow cytometer was unavailable to us during the initial experiment, but subsequently we grew up the isolates of each species to make a dilution series and constructed calibration curves to estimate cell counts from OD measures (electronic supplementary material, figure S1). We present the results using calibrated cell counts per ml here, but results were qualitatively the same using raw OD: more of the variation in OD measures was due to idiosyncratic species responses than in the calibrated counts, but the general trends with species richness remained the same as reported here.

We fitted linear models to growth rates and yields (and their change relative to ancestral isolates) as response variables, with environment (beech, pH5 or spruce), richness and (in some cases) composition as explanatory variables. Models were simplified using stepwise ANOVA to obtain minimum adequate models. To control for the possible effect of species sorting on trends in growth rates and yields with diversity, we fitted linear mixed-effects models with random intercepts and slopes with log(diversity) for each species in each environment: this treats a given species × environment combination as a block, and consistent trends with richness across multiple combinations are required for a significant fixed effect. To partition variation into different sources, the proportion of variance explained by sets of terms and their interactions was calculated from full models without simplification, and we successively added in further sets of explanatory variables to calculate the reduction in residual deviance caused by each set. All analyses were conducted in the R statistical programming language and using the ‘lme4’ and ‘lmerTest’ packages for mixed-effects models [31,32].

## 3. Results

### (a) Growth rates and yields of monoculture isolates

**Figure 2.**
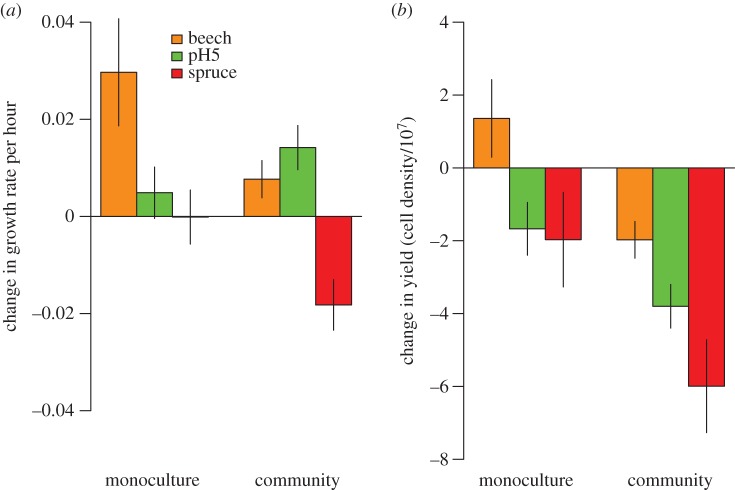
The change in (*a*) growth rate and (*b*) yields in monoculture and community isolates minus the growth rate and yields of ancestral isolates in each environment, respectively. Note that growth rates were calculated as log(cells per ml at 24 h/cells per ml at 0 h)/24 h, and hence have units of per hour. Growth rates increased on average in monoculture isolates, but increased less or decreased in community isolates. Yields did not change in monocultures, but declined in community isolates, especially in pH5 and spruce tea.

Most species survived in monoculture: only one species from spruce tea was not recoverable. As predicted, monoculture isolates evolved significantly faster growth rates on average than ancestral isolates (figure 2*a*; linear model of growth rate of monoculture minus growth rate of ancestor, intercept = 0.012, *t* = 2.54, d.f. = 103, *p* = 0.013). Responses varied across environments. Growth rates increased more in beech tea than in pH5 tea or spruce tea (figure 2*a*; effect of environment, *F*_2,101_ = 4.1, *p* = 0.020), contrary to the prediction that growth rates should increase most in the environment causing the greatest initial decline. Responses did not vary significantly among species (*F*_2,90_ = 1.1, *p* = 0.40); hence, this term was removed from the model. As predicted, however, the change in growth rate was greater for monocultures with initially lower ancestral rates (slope = −0.30, *t* = −3.89, d.f. = 100, *p* = 0.00018), with no variation in slope across environments (interaction term *F*_2,98_ = 0.05, *p* = 0.95).

Yields did not change on average between monoculture and ancestral isolates (figure 2*b*; linear model of monoculture yield minus ancestral yield, intercept = −7.1 × 10^6^, *t* = −1.17, d.f. = 103, *p* = 0.25). The change varied marginally across treatments, being positive in beech tea and negative in pH5 tea and spruce tea (*F*_2,101_ = 3.2, *p* = 0.045). Whether yields increased or decreased in a particular environment varied considerably among species (electronic supplementary material, figure S2; *F*_21,69_ = 3.3, *p* < 0.0001). Changes in yield were greater in species with lower ancestral yields in spruce and pH5 tea, but not beech tea (*F*_2,98_ = 19.5, *p* < 0.0001). There was no evidence for a negative trade-off between changes in growth rates and changes in yields in any environment (third row in electronic supplementary material, figure S3; *F*_2,98_ = 0.66, *p* = 0.52).

### (b) Species interactions and extinction in community microcosms

Species interactions in the communities were generally negative, as indicated by the densities of community microcosms being significantly lower than the sum of the densities of constituent species in monoculture [27]. Of 225 community microcosms at the start, 72% displayed antagonistic interactions (community yield was less than the maximum yield of the monocultures), 24% displayed partial complementarity (community yield was higher than the maximum yield of the monocultures but less than the sum of monoculture yields, i.e. interspecific interactions were negative but weaker than intraspecific interactions) and 4% were synergistic (community yield exceeded the sum of monoculture yields; electronic supplementary material, figure S4). As predicted, species extinction rates increased dramatically with species richness (e.g. from zero in monocultures to 74% in 12-species communities on control beech tea [27]; electronic supplementary material, figure S5). Nonetheless, across compositions and environments, final diversity still correlated significantly with starting diversity (*t* = 3.5, d.f. = 24, *p* = 0.0017, *R*^2^ = 0.34). Extinction rates were higher in pH5 and spruce tea than in control beech tea (e.g. 84% and 86%, respectively, in 12-species communities; d.f. = 326, both *z* > 3, *p* < 0.005, generalized linear model with binomial errors). Most variation was explained by species identity, richness and their interaction (explained deviance = 20.3%, 20.6%, 11.9%, respectively, all *p* < 0.0001). As predicted, extinction risk was higher for species with lower ancestral growth rates (GLM: *z* = −5.8, *n* = 864, *p* < 0.0001) and less strongly with lower yields (*z* = −3.2, *p* = 0.0012), although these explained far less deviance (3.7% and 0.9%, respectively) than species identity and richness.

### (c) Growth rates and yields of community isolates

Surviving species that evolved in communities had different growth characteristics than the same species evolving in monoculture, in ways that varied among environments (figure 2*a*). Whereas growth rates increased, on average, in monoculture isolates across all environments, there was no overall trend in community isolates (linear model of community isolate minus ancestor growth rate, intercept = 0.002, *t* = 0.81, d.f. = 320, *p* = 0.42). Growth rates of isolates from communities were no longer significantly faster on average than ancestors in beech tea (*t* = 1.90, d.f. = 318, *p* = 0.059) and were significantly slower than ancestors in spruce tea (*t* = −4.14, d.f. = 318, *p* < 0.0001). These results match predictions if competition led to specialization on fewer or less rewarding resources than in monocultures. In contrast, species interactions had no effect on the response in pH5 tea: growth rates of isolates in pH5 tea evolved to be significantly faster than ancestors in communities, just as they had in monocultures (effect of monoculture versus community in pH5 tea, *t* = 1.14, d.f. = 132, *p* = 0.26; green bar in figure 2*a*). The relationship between the change in growth rate and ancestral growth rate was still negative (slope = −0.22, *t* = −5.57, *p* < 0.0001; electronic supplementary material, figure S3), as observed in monocultures.

Although yields did not change consistently in monocultures, yields of community isolates declined on average (figure 2*b*; *t* = −7.91, d.f. = 320, *p* < 0.0001), especially strongly in spruce tea (*t* = −3.59, d.f. = 318, *p* = 0.0004). Species still varied significantly in their response to each environment (electronic supplementary material, figure S2; *F*_18,289_ = 5.47, *p* < 0.0001). The relationship between the change in yield and ancestral yield was more negative among community isolates (electronic supplementary material, figure S3; slope relative to slope in monocultures, −0.18, *t* = −4.0, d.f. = 421, *p* < 0.0001): isolates with high ancestral yields evolved lower yields in communities than in monocultures. There was no trade-off between change in growth rate and change in yield, similarly to the findings in monocultures (*t* = 0.9, d.f. = 319, *p* = 0.42).

We tested for potential divergence of species in a community into high growth rate (low yield) and high yield (low growth rate) specialists. We measured whether the range of growth rates and yields in turn was greater between evolved isolates versus the ancestral isolates of those species, for each community. Across environments, instead of divergence, there was significant convergence of both growth rates (paired *t*-test, mean difference = −0.025, d.f. = 90, *t* = −3.64, *p* = 0.0005) and yields (paired *t*-test, mean difference = −6.2 × 10^7^, *t* = −6.27, *p* < 0.0001). This matches the finding of negative relationships between changes and starting values: under divergence, we would expect a positive relationship as extreme values become more extreme.

### (d) Variation in responses with species richness

**Figure 3.**
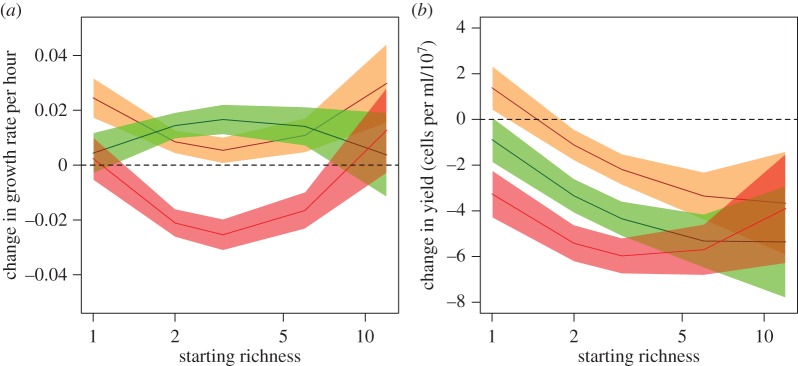
Changes in growth rates and yields relative to ancestors across environments and richness levels. (*a*) Growth rates against starting richness. (*b*) Yield against starting richness. Lines show the average trend across species in each environment from linear models including interactions between log(richness), log(richness)^2^ and environment (electronic supplementary material, table S3).

Changes in growth rates and yields relative to ancestral isolates varied significantly with the starting richness of the community (figure 3; electronic supplementary material, figure S6). Growth rates in beech and spruce tea showed U-shaped responses: they were lower in two- to six-species cultures, but similar in 12-species cultures to monocultures, (figure 3*a*; slope with quadratic log(richness) = 0.0137, *t* = 2.4, d.f. = 387, *p* = 0.017). Growth rates in pH5 tea did not vary significantly with species richness. Note that these trends are found controlling for species identity as random effects (electronic supplementary material, table S3), and therefore did not simply reflect differential survival of species but rather a general trend in responses across species and treatments. Yields in beech tea and pH5 tea declined with increasing richness (figure 3*b*; *t* = −3.66, *p* = 0.0003), whereas yields in spruce tea showed a U-shaped response: declining at intermediate richness but unchanged compared with ancestors in monocultures and in 12-species cultures (slope with log(richness)^2^ = 1.51 × 10^7^, *t* = 2.4, d.f. = 23, *p* = 0.025). Final richness of surviving species did not explain further variation in any environment (ANOVAs comparing the best model for starting richness versus a model including an interaction between final richness and environment: for growth rate, *χ*^2^ = 6.85, d.f. = 3, *p* = 0.077; for yields, *χ*^2^ = 1.52, d.f. = 3, *p* = 0.68).

We partitioned variation in growth traits into the effects of starting richness, final richness, species identity and community composition (table 1). Interactions between focal species identity and environment irrespective of species richness explained 36.5% of deviance in changes in growth rate and 56.8% for changes in yield (electronic supplementary material, figure S6 shows separate plots for each species). Adding in general effects of starting richness in each environment explained a further 3.0% for changes in growth rates and 3.4% for changes in yield. Adding in general effects of final richness explained little more: 0.9% and 0.0%, respectively. Allowing the effects of species richness to vary among focal species as well as environments explained 16.6% more deviance in growth rates and 10.4% more for yields. Composition of the background community explained a further 6.9% and 3.9% (not significant; table 1), and the remainder represents residual variation among replicates within a given combination of species by environment by composition. Of 24.6% and 17.4% of deviance in changes in growth rates and yield explained by focal species and its interactions with other variables, 13.3% and 11.8%, respectively, was explained by ancestral growth rates and yields of each species in turn.

**Table 1.** Analysis of variance showing the cumulative variation explained by adding successive terms into the model. *S* = focal species, *E* = environmental treatment, *RS* = log(starting richness), *RF* = log(final richness), *C* = community composition coded as a factor. *F*-values and *p*-values refer to ANOVA comparing model with and without those terms added.

response	terms added	cumulative % deviance explained	*F*	*DF*1	*DF*2	*p*
growth rate	*S*	0.155	6.86	11	413	<0.0001
	E*S	0.365	5.61	23	390	<0.0001
	E*(*RS* + *RS*^2^)	0.395	3.19	6	384	0.0045
	E*RF	0.404	1.91	3	381	0.13
	E*S*(*RS* + *RS*^2^ + *RF*)	0.569	1.98	62	319	0.0001
	E*C	0.638	1.02	50	269	0.44
yield	*S*	0.430	28.35	11	413	<0.0001
	E*S	0.568	5.43	23	390	<0.0001
	E*(*RS* + *RS*^2^)	0.603	5.53	6	384	<0.0001
	E*RF	0.603	0.20	3	381	0.90
	E*S*(*RS* + *RS*^2^ + *RF*)	0.707	1.83	62	319	0.0004
	E*C	0.747	0.84	50	269	0.78

## 4. Discussion

Species interactions altered the evolution of growth rate and yield of constituent species, as measured by growth of isolates extracted from their communities. Although growth in isolation does not directly reflect growth in the presence of the other species (i.e. the context that each isolate evolved in), it provides a tractable assay for a large number of isolates that can be interpreted in terms of altered growth phenotypes due to species interactions, as follows. In monocultures, as expected for serial transfer experiments, isolates evolved increased growth rate on average across environments. Our media contained a complex chemical mixture in beech and spruce tea supplemented with R2A ingredients. Species could have evolved higher growth rates by specializing on more energetically rewarding substrates or by shifting to higher-rate metabolic pathways. Our previous work showed that resource use converged in monocultures of four species studied here [33]. There was no evidence for a negative trade-off between growth rates and yields in any environments, contrary to predictions of theory [13,14].

The predominantly negative interactions we observed in these communities reduced the evolution of faster growth rates of species that survived in beech tea and spruce tea. One possible explanation is that species specialized to use distinct resources in the tea, which would lead to lower growth rates compared with monoculture isolates that adapted to using more resources. This explanation is supported by previous evidence for one community of four of the species studied here: species diverged in resource use when they evolved together in a community [33]. We found no evidence for a negative trade-off between changes in *r* and the yield or that species specialized as high-*r* versus high-yield strategists: instead species tended to converge towards similar values in community microcosms. Other explanations for altered growth rates would be if species allocated resources away from growth towards direct interactions with other species, such as the production of biocides [34], or if species lost energetically costly detoxification or resource acquisition mechanisms (e.g. siderophores) because they were provided by other species in the mixtures [35]. Our design mimics cases with regular arrival of new resources and dilution of standing populations, such as might occur in gut microbiomes or periodically disturbed intertidal communities. Outcomes might vary depending on the supply rate and predictability of resource inputs, as shown by evolution experiments with two species of bacteria cultured with fluctuating resources [36].

The effects varied with species richness and the number of interactions. Even though extinction rates were relatively high, starting richness provided a better explanation of trends than final richness. The evolutionary trajectory of surviving species was altered even in microcosms with low final diversity. The general relationships with richness either saturated or reversed at the highest levels of richness, which confirms predictions that the effects of species interactions average out or weaken when more species are present in the community. This finding mirrors inferences in other systems. For example, in forests, strong pairwise interspecific associations between tree species decline from low diversity temperate forests through to species-rich tropical forests [37], which has been argued to favour evolutionary convergence towards competitive equivalence [38]. This prediction is potentially supported for microbial communities by the observation of generally negative interactions between wild isolates [18], implying generally overlapping resource uses at least as assayed in simplified laboratory environments. Similarly, diffuse coevolution with multiple herbivore species reduced the evolution of resistance in *Solanum carolinense*, because of genetic covariance in resistance to alternative herbivore species feeding on different plant structures [39].

Despite the general trends, more variation resulted from species-specific responses than from general effects of richness across species, with over half of the variation explained by focal species being explained by their ancestral growth rates. It therefore remains possible that particular pairwise interactions exert a strong effect on some species even in more diverse systems.

## 5. Conclusion

Our results show the importance of community context for determining evolutionary responses, in line with growing number of studies. Isolates that evolved in communities had slower growth rates and lower yields than monoculture isolates, consistent with recent theory that competition constrains species to specialize on narrower sets of resources. However, these effects became saturated or reversed at higher levels of diversity: the biggest overall negative changes occurred in comparison of two-species cultures relative to monocultures. Mechanistic models and experimental systems are now needed for predicting species responses in diverse systems. Our experiments considered small communities relative to wild bacterial communities. New methods of tracking evolution and species interactions in mixtures are needed to infer interaction networks and to determine whether strong pairwise interactions shape evolution in natural bacterial communities with many hundreds of co-occurring species.

## Supplementary Material

Supplementary Material

## Data Availability

Datasets and code are available from Dryad: http://dx.doi.org/10.5061/dryad.79gq3.
