# Supplementary material for: Saturating effects of species diversity on life-history evolution in bacteria
Source: Proc Biol Sci. 2015 Sep 22;282(1815):20151794. doi: 10.1098/rspb.2015.1794 (PMC4614762; doi:10.1098/rspb.2015.1794)

Supplementary Material – Fiegna et al. 'Saturating effects of species diversity on life-history evolution in bacteria'.

Table S1. Species isolates used in the experiments.

Isolate	Putative genus/species	Family	Phylum	Colony phenotype
THB2	*Mucilaginibacter sp.*	Sphingobacteriaceae	Bacteroidetes	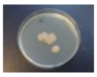
THB6	*Flavobacterium sp.*	Flavobacteriaceae	Bacteroidetes	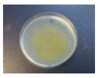
THB7	*Flavobacterium sp.*	Flavobacteriaceae	Bacteroidetes	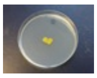
THB9	*Sphingomonas faeni*	Sphingomonadaceae	Alphaproteobacteria	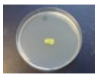
THB14	*Yersinia ruckeri*	Enterobacteriaceae	Gammaproteobacteria	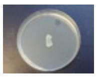
THB18	*Pseudomonas veroni*	Pseudomonadaceae	Gammaproteobacteria	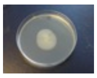
THB20	*Pseudomonas sp.*	Pseudomonadaceae	Gammaproteobacteria	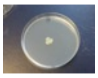
THB22	*Pseudomonas trivialis*	Pseudomonadaceae	Gammaproteobacteria	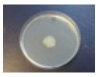
THB29	*Novosphingobium sp.*	Sphingomonadaceae	Alphaproteobacteria	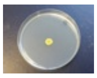
THB32	*Chryseobacterium sp.*	Flavobacteriaceae	Bacteroidetes	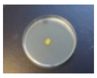
THB39	*Rhodococcus sp.*	Nocardiaceae	Actinobacteria	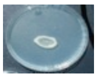
THB63	*Pedobacter sp.*	Sphingobacteriaceae	Bacteroidetes	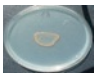

Table S2 - Composition of the experimental communities. Species numbers refer to numbers in table 1, e.g. 2,6 indicates that THB2 and THB6 were present. Composition Y had all 12 species together. Monocultures were labelled by their species number.

Composition	Species	Composition	Species
A	2,6	M	2,22,29
B	22,39	N	9,18,39
C	29,63	O	7,20,63
D	9,18	P	6,14,32
E	14,32	Q	22,32,63
F	7,20	R	2,7,18
G	7,18	S	14,20,29
H	14,20	T	6,9,39
I	2,29	U	9,14,18,20,29,32
J	9,63	V	2,6,7,22,39,63
K	6,22	W	6,7,9,18,32,63
L	32,39	X	2,14,20,22,29,39

Table S3. Linear mixed effects models of growth rates and yields against starting richness and final richness. Each model was simplified using stepwise removal from the maximum model and ANOVA.

A) Growth rates

Fixed effects	Estimate	Standard Error	Degrees of freedom	t	p
Intercept	0.0246	0.0091	50.3	2.70	0.0094**
log(richness.start)	-0.0360	0.0128	393.3	-2.81	0.0052**
pH5	-0.0200	0.0129	50.8	-1.56	0.1262
spruce	-0.0228	0.0132	51.4	-1.73	0.0900
$\log(\text{richness})^2$	0.0137	0.0057	386.6	2.40	0.0169*
log(richness.start):pH5	0.0431	0.0188	400.2	2.29	0.0228*
log(richness.start):spruce	-0.0061	0.0195	403.4	-0.31	0.7537
$\log(\text{richness.start})^2$:pH5	-0.0189	0.0084	388.9	-2.25	0.0249*
$\log(\text{richness.start})^2$:spruce	0.0064	0.0086	398	0.75	0.4542

Random effects	Variance
Species x Environment: intercept	5.25E-04
Species x Environment: Slope	2.72E-06

B) Yields

	Estimate	Standard Error	Degrees of freedom	t	p
Intercept	1.123	1.591	40.4	0.71	0.4843
log(richness.start)	-4.043	1.105	261.4	-3.66	0.0003
pH5	1.358	0.593	113.1	2.29	0.0239
spruce	-2.166	2.169	38.2	-1.00	0.3243
$\log(\text{richness.start})^2$	-3.794	2.223	38.5	-1.71	0.0960
$\log(\text{richness})^2$:pH5	-0.287	0.624	24.6	-0.46	0.6501
$\log(\text{richness})^2$:spruce	1.508	0.628	22.7	2.40	0.0250

Random effects	Variance
Species x Environment: intercept	2.70E+15
Species x Environment: Slope	3.76E+14

FIGURE S1. Calibration curves between flow cytometer cell counts (y-axis) and OD measures (x-axis) from repeated dilution series of each species. To calibrate OD data into cell counts we grew isolates for three to four days in R2A supplemented beech tea buffered to pH5 and pH7 until OD measurements were suitable. Every isolate was diluted in sterilized medium into six different concentrations from 100% density down to 3% density and a final volume of 200 µl. From each dilution 150 µl were transferred into a clear 96-well plate and optical density was determined at 595nm (OD). In parallel 2 µl of sample were further diluted in 198 µl milli-Q (Millipore) water and subsequently 33 µl were analysed on a BD Accuri™ C6 flow cytometer. Appropriate gating based on the SSC was designed to exclude debris from the media. OD was transformed into cell counts using linear regression models fitted to each species separately.

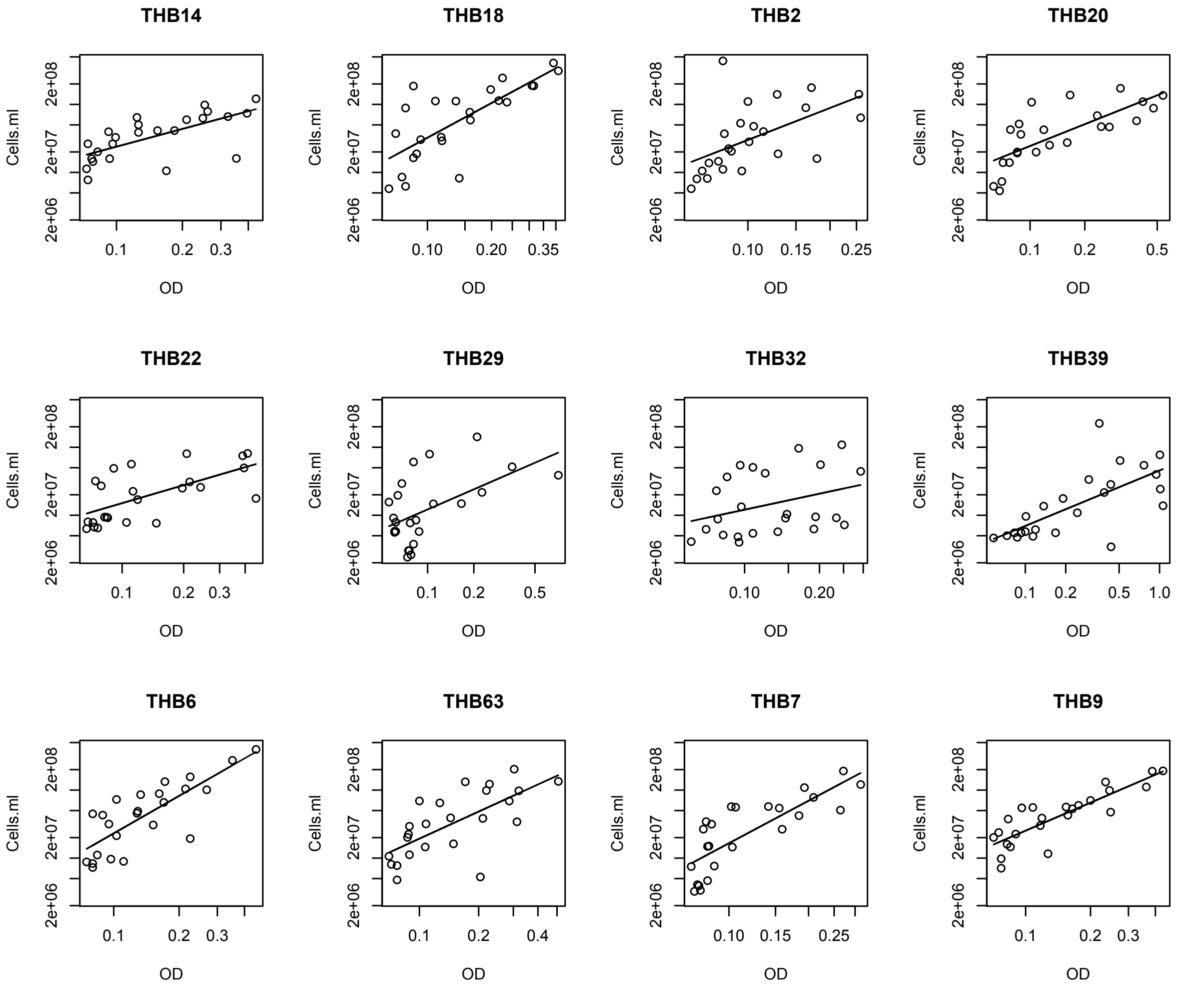

FIGURE S2. Changes in growth rates (units of per hour) and yields across species in monocultures (circles, solid lines) and communities (crosses, dotted lines). Orange=beech tea; green = pH5 tea; red = spruce tea. The isolate reference number for each species is shown.

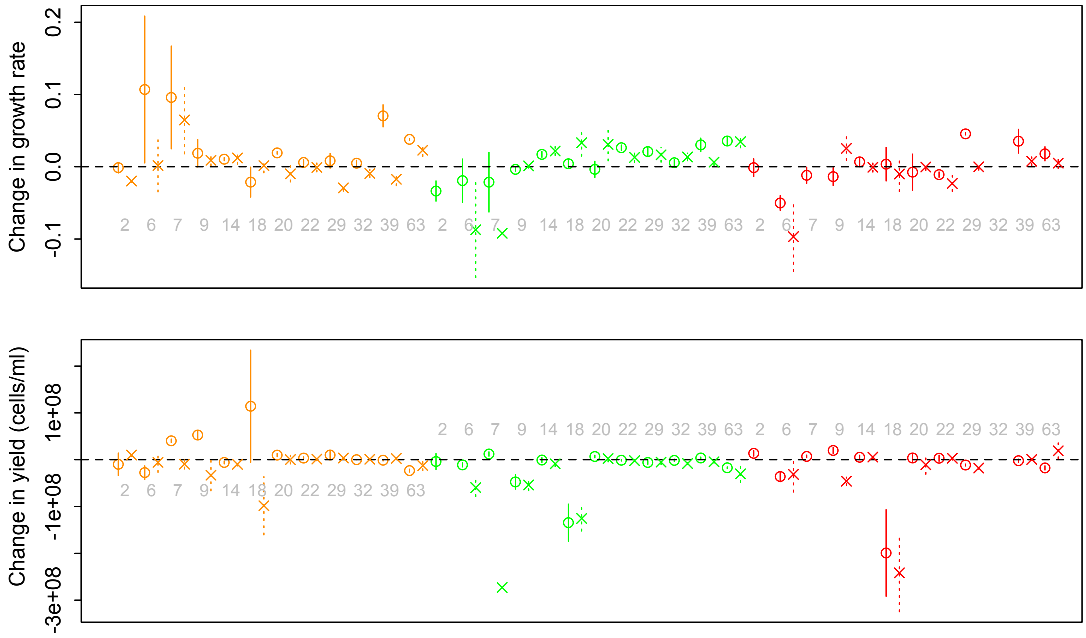

FIGURE S3. Evolutionary trajectories of changes in each environment. Top row: Change in growth rate plotted against ancestral growth rate. Dotted line=monoculture regression line; solid line = communities regression line. Second row: change in yield against ancestral yield. Bottom row: change in growth rate against change in yield; a negative slope would indicate a negative trade-off between growth rate and yield.

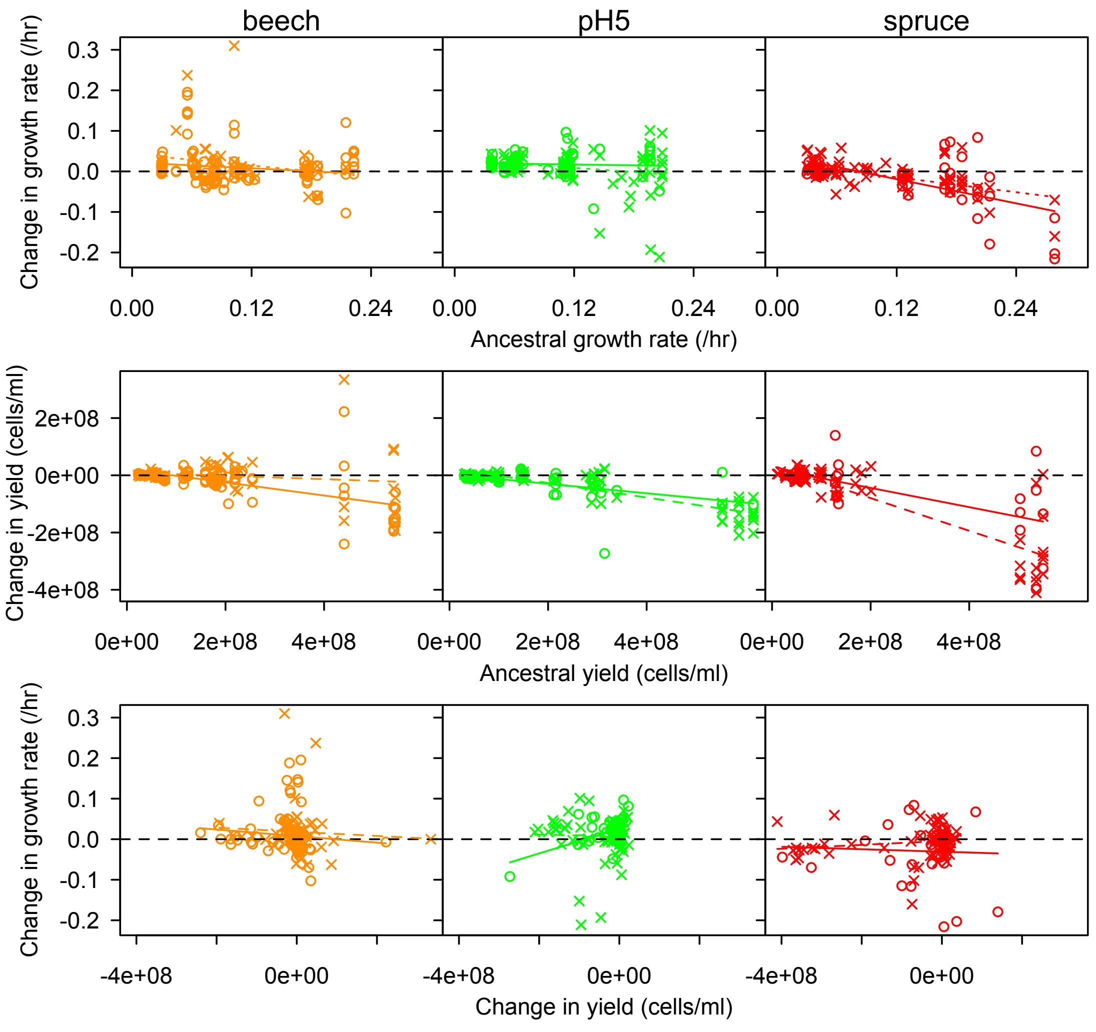

FIGURE S4. Observed yields (units = cells per ml) of community microcosms was lower than the sum of the yields of the monocultures of each species contained in the community. A 1:1 relationship is expected if there are no species interactions and combined yield is additive. Instead, the yield of communities was on average 40.6%, 47.4% and 59.4% of the predicted additive yield in beech tea, pH5 tea and spruce tea respectively. Yields expressed as OD units to match Fiegna et al. 2015 are shown.

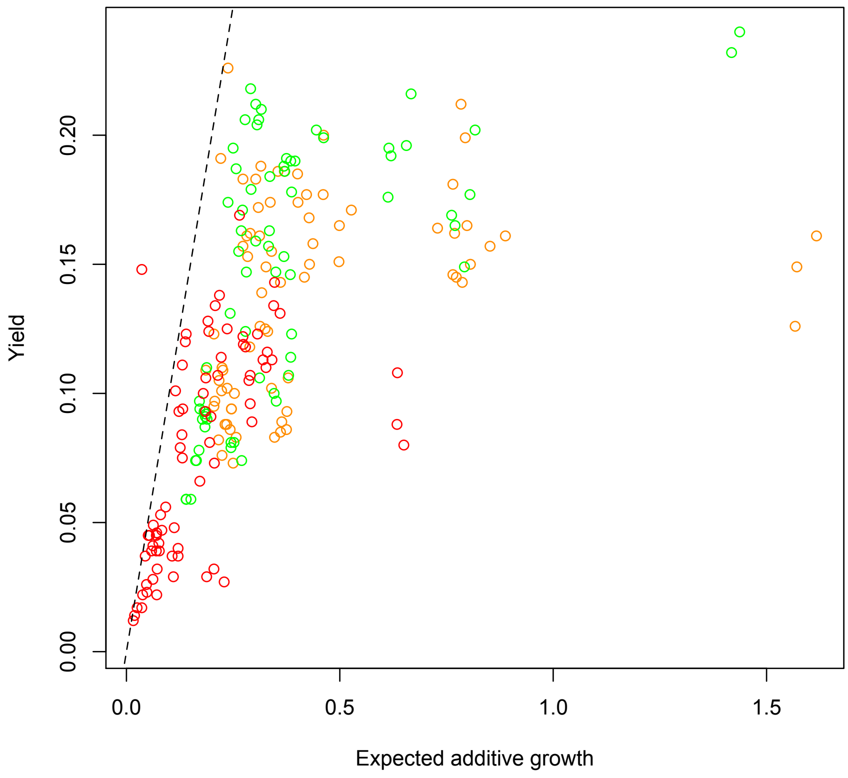

FIGURE S5. The per species probability of extinction during the experiment in relation to the starting richness of the community and the environment. Extinction was recorded as the failure to recover a species from the community at the end.

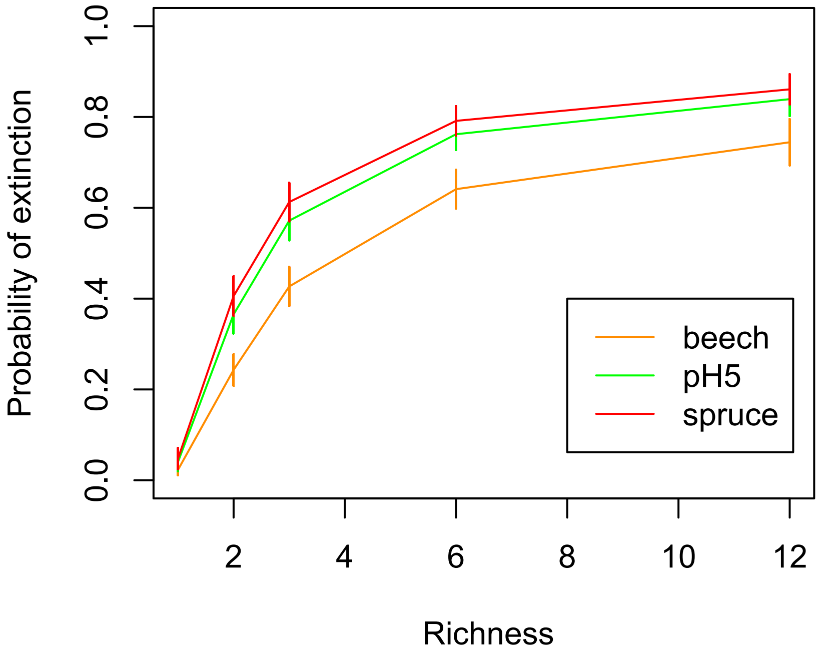

FIGURE S6. Fitted relationships between A) species growth rates (units of per hour) and B) species yields (units of cells per ml) with environment and species richness. Each panel represents a separate species. The x-axis is shown in log scale.

A)

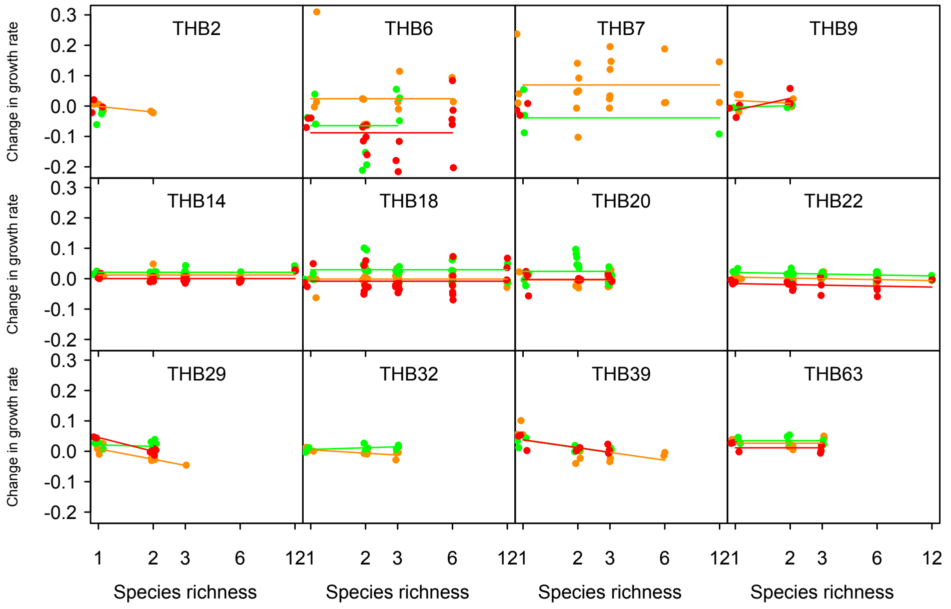

B)

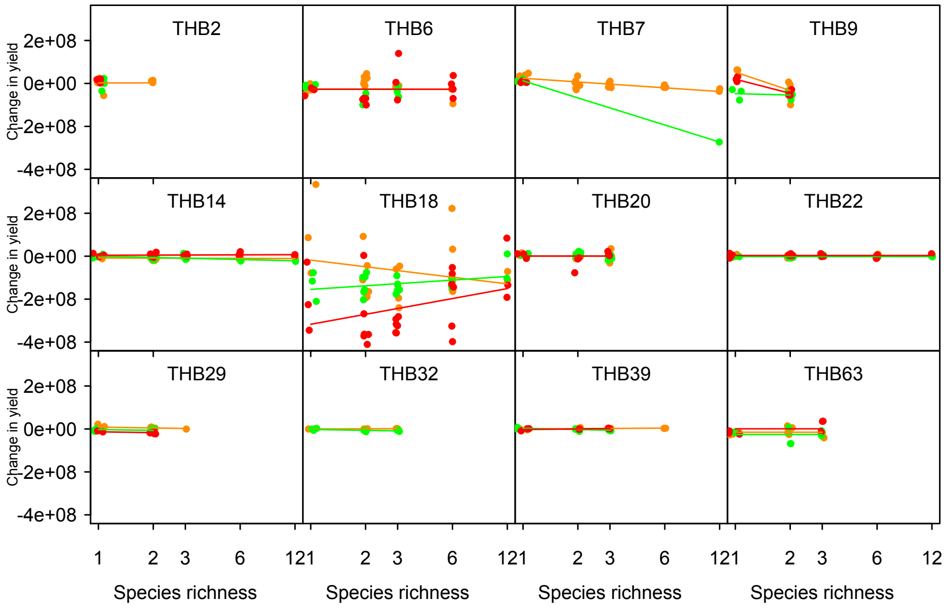

Supplement: Supplementary Material [file rspb20151794supp1.pdf]
